# Formulation of chelating agent with surfactant in cloud point extraction of methylphenol in water

**DOI:** 10.1098/rsos.180070

**Published:** 2018-07-04

**Authors:** H. Z. Hazrina, M. S. Noorashikin, S. Y. Beh, S. H. Loh, N. N. M. Zain

**Affiliations:** 1 School of Marine Science and Environment, Universiti Malaysia Terengganu, 21030 Kuala Terengganu, Terengganu, Malaysia; 2 Chemical Engineering Programme, Research Centre For Sustainable Process Technology (CESPRO), Faculty of Engineering and Built Environment, Universiti Kebangsaan Malaysia, 43600 UKM Bangi, Selangor, Malaysia; 3 Integrative Medicine Cluster, Advanced Medical and Dental Institute, Universiti Sains Malaysia, Bertam, 13200 Kepala Batas, Pulau Pinang, Malaysia

**Keywords:** methylphenol, cloud point extraction, additives, high-performance liquid chromatography, non-ionic surfactant

## Abstract

Cloud point extraction (CPE) is a separation and preconcentration of non-ionic surfactant from one liquid phase to another. In this study, Sylgard 309 and three different types of additives for CPE, namely CPE-Sylgard, CPE-Sylgard-BMIMBr and CPE-Sylgard-GLDA, are investigated to extract methylphenol from water samples. The methylphenols are well separated by reversed-phase high-performance liquid chromatography (HPLC) with isocratic elution of acetonitrile : water; 60 : 40 (v/v) and detection at 260 nm. The optimized parameters for the effect of salt, surfactant, temperature, time of extraction, pH, interference study and the performance of different additives on methylphenol extraction are investigated. CPE-Sylgard-GLDA is chosen because it gives us a high peak and good peak area compared with CPE-Sylgard and CPE-Sylgard-BMIMBr. The recovery extractions of CPE-Sylgard-GLDA are obtained in the range of 80–99% as the percentage of relative standard deviation (RSD) is less than 10. The LOD and LOQ are 0.05 ppm and 0.18 ppm, respectively. The method developed for CPE-Sylgard-GLDA coupled with HPLC is feasible for the determination of methylphenol because it is simple, effective, cheap, and produces a high percentage of recovery.

## 1. Introduction

Phenols, one of the hazardous pollutants, exist extensively in the environment. The phenols in the environment mainly come from industrial and agricultural products such as drugs, dyes, pesticides and paper. Besides that, phenols are also formed from natural processes such as decomposition of leaves and wood [1]. However, the anthropogenic activities can be considered as the main source of phenols in the environment [2].

Methylphenol is one of the commonly detected pollutants in water because it is widely used in many industrial processes. It is an organic compound, which is bonded directly to a hydroxyl group and has limited solubility in water (8.3 g/100 ml). It is also very toxic and has a diverse effect on the taste and odour of water at low concentration and low biodegradability. The compound exists as a waste product during the production of medicines, pesticides, perfumes, photographic film developer, dyes and in the petrochemical industry. In Malaysia, the acceptable limit of methylphenol concentration in wastewater is 0.001 mg l^−1^. The European Community (EC) Directive specifies the legal tolerance level of 0.5 µg l^−1^ for the phenol in water intended for human consumption.

Because of the widespread use of phenolic compounds around the world and their toxicity, which can cause several chronic effects such as endocrine disruption, hepatoxicity and neurotoxicity in humans and other organisms even at environmental levels [3], these compounds are listed as top pollutants by the US Environmental Protection Agency (EPA) and the European Union [2]. Most phenolic compounds can easily penetrate the skin and can readily be absorbed in the human gastrointestinal tract. Once they enter the system, phenolic compounds undergo metabolism and transform into various reactive intermediate forms particularly quinone moieties, which can easily form covalent bonds with proteins, exerting toxic effects on humans [4]. As a consequence of the large use of phenolic compounds, allied to their high toxicity and mobility in soil, the analytical methods for phenolic compound determination in water and soil are of prime importance.

Several separation and preconcentration methods such as liquid-phase microextraction (LPME), dispersive liquid–liquid microextraction (DLLME), solid phase extraction (SPE) and cloud point extraction (CPE) have been widely used in analytical chemistry. The CPE technique is one of the most popular sample pretreatment approaches. This extraction technique is based on the mixing of either cationic/anionic surfactant with non-ionic surfactants and formation of covalent hydrophobic chelates of the respective metal ion with suitable reagents. However, this method has some limitations. The SPE method consumes large sample volumes and toxic organic solvents and requires prolonged steps, which make it labour-intensive, expensive and environmentally unfriendly. On the other hand, the limitation of LPME and DLLME is the absorption of the macromolecule to the surface of the coating, negatively affecting the yield of extraction. Meanwhile, CPE offers many advantages because it is cost effective, easy, rapid etc. regarding the sample preparation [5]. These techniques also overcome the few drawbacks of the conventional techniques [6].

CPE is the process of separation and preconcentration of non-ionic surfactant from one liquid phase to another [7]. The technique is based on the micellar solution of non-ionic surfactant cloudy forms that separates into a concentrated phase containing most of the surfactants, known as the surfactant-rich phase [8,9]. The homogeneous aqueous solution of non-ionic surfactant becomes turbid and phase separation occurs when heated beyond the temperature known as the cloud point temperature (CPT) [10]. Specifically, the micellar solution separates into a surfactant-rich phase and dilute aqueous phase [11].

In recent times, CPE has become one of the most preferred methods used for the separation technique [12]. CPE promises the principle of a green chemistry method because it uses cheap and low-cost, non-toxic surfactants, and because of its non-volatility, high selectivity and high enrichment factor with high recoveries. The effectiveness of the CPE technique can avoid hazardous organic solvents.

The CPE can be improved by changing the micellization of the surfactant, and increasing the solubility of micelles, temperature and additive content [13]. According to Gao *et al.* [9] and Bhatt *et al.* [14], few studies have reported the use of ionic liquids (ILs) as an additive with non-ionic surfactants in CPE [9,14]. In this study, 1-butyl-3-methylimidazolium bromide (BMIMBr), an IL, and tetrasodium of *N*, *N*-bis(carboxymethyl) glutamic acid (GLDA), a chelating agent, are used as additives.

ILs, sometimes known as molten salt, are widely recognized for use in analytical chemistry [15], including extraction in gas chromatography (GC), in liquid chromatography (LC) and in capillary electrophoresis (CE), because they possess several unique properties. ILs comprise organic cations such as imidazolium, pyridinium, pyrrolidinium, phosphonium and ammonium which when paired with a variety of anions acquire most of the properties of conventional organic solvents. Their unique properties, such as wide liquid temperature range, low melting points and negligible vapour pressure, have triggered research in exploring their use as a replacement for the traditionally more toxic, flammable and volatile organic solvents. ILs provide an alternative medium for ‘green chemistry’ owing to their negligible vapour pressure [15,16].

Meanwhile, a chelating agent is widely used in the extraction of heavy metals. The pioneer work on the removal of heavy metal ions was initiated by Dudzinska and Juang by using complex agents such as nitrilotriacetic acid (NTA), citric acid (CA) and ethylenediaminetetraacetic acid (EDTA) [17].

The carbon content of EDTA is fossil-based, whereas the carbon source of GLDA is primarily biobased. Therefore, GLDA is the only chelating agent with ‘green’ carbon atoms. The biodegradation of GLDA is initiated by mono-oxygenases catalysing the removal of the carboxymethyl group. According to the Swedish Society for Nature Conservation, GLDA is 86% based on natural raw material. It also possesses good solubility at low and high pH, while greater than 60% of l-GLDA degrades within 28 days. The Dissolvine GL-38 contains only the l-form. This is significant because the d-form is non-biodegradable [17–19]. The thermal stability of GLDA is surprisingly high. When tested at a temperature above 573 K, it showed no significant decomposition. This property has been used to develop water treatment systems for operating boilers to reduce the effect of hard water.

Several tests have also shown that GLDA has up to 10 times higher solubility in 25% sodium hydroxide solution (NaOH) compared to EDTA and NTA [19]. GLDA also has been characterized as having an excellent solubility at low pH [17,20]. Another notable feature is that the ecological footprint of GLDA is far smaller than those from the traditional counterparts because of its efficient manufacturing processes. Previous studies with regard to the usage of GLDA have centred on its applications in detergents, cosmetics and boosting agents for disinfectants [17,18].

In this research, the cloud point extraction process using Sylgard 309 non-ionic surfactant and additives (GLDA, BMIMBr) to extract methylphenol compounds from water samples is investigated. The selected parameters are the salt concentration, surfactant, extraction time and temperature. These parameters will lead to the achievement of high percentage recoveries of methylphenol.

## 2. Experimental design

### 2.1. Reagent and standard

Sylgard 309 non-ionic surfactant was purchased from Dow Corning (Shanghai, China) supplied by Dow Corning Malaysia. Methylphenol liquid was purchased from Sigma Aldrich (Germany). Acetonitrile (HPLC grade) was purchased from Merck (Germany) and the deionized water used in the mobile phase was of conductivity at 18 MΩ cm. Sodium sulphate salt, Na_2_SO_4_, was obtained from Merck (Germany) and stock solutions of methylphenol at a concentration of 1000 mg l^−1^ were prepared in acetonitrile. Working standard solutions were prepared by step-wise dilution with deionized water of the stock solutions. The pH of the solution samples was adjusted with diluted hydrochloric acid or diluted sodium hydroxide solutions. The apparatus and glassware used included beakers with different size (100 ml, 200 ml), micropipette, volumetric flask, test tube, centrifuge tube (15 ml), spatula, syringe, vials and reagent bottles with different size (250 ml, 500 ml).

### 2.2. Instrumentation

The separation and quantification of the tested methylphenol were carried out with a Shimadzu HPLC system. The system consisted of a pump, degasser, auto injector, column oven, ultraviolet detector, guard column and Chromolith C18 column (100 mm × 4.6 mm, Merck, Germany). HPLC isocratic conditions were used to separate the analytes using acetonitrile and deionized water at flow rates of 1 ml min^−1^ and detection at 280 nm.

### 2.3. Cloud point extraction procedure

In the general procedure for the CPE method, 0.2 ml of deionized water, 0.2 ml 15% additives of GLDA and BMIMBr, 0.2 ml 15% (w/v) surfactant aqueous solution for non-additives and GLDA, 0.2 ml 20% (w/v) surfactant aqueous solution for BMIMBr, 2.5 ml of stock solution of methylphenol and 0.5 ml 1 M Na_2_SO_4_ were added into a 15 ml centrifuge tube. The mixture of the solution was sonicated using an ultrasonicator for about 5 min to obtain the desired formation of the two layer phases, which are the surfactant-rich phase and aqueous solution. The volume of the surfactant-rich phase and water content were measured using the centrifuge tube scale. The top layer of the phase was separated with a syringe into vials and was directly injected into the HPLC system for analysis. All data were conducted in triplicate.

### 2.4. Optimization of parameters for methylphenol extraction

#### 2.4.1. Effect of salt concentrations

The extraction of methylphenol was conducted in different concentrations of salt (0.5 M, 1.0 M, 1.5 M, 2.0 M and 2.5 M), while other parameters were kept constant.

#### 2.4.2. Effect of silicone non-ionic surfactant (Sylgard 309) concentrations

A series of non-ionic surfactant Sylgard 309 samples was prepared at various concentrations from 5% (w/v) to 40% (w/v) using deionized water, while other parameters were kept constant.

#### 2.4.3. Effect of additives

Series of additives (GLDA & BMIMBr) were prepared at various concentrations, 5% (w/v), 10% (w/v), 15% (w/v), 20% (w/v), 30% (w/v) and 40% (w/v), using deionized water, while other parameters were kept constant.

#### 2.4.4. Effect of temperature

The extraction of methylphenol was carried out in a water bath in order to ensure the desired temperatures (27°C, 35°C, 45°C, 55°C and 65°C) were achieved while other parameters were kept constant.

#### 2.4.5. Effect of pH

A series of samples at different pH values (2, 4, 6, 7, 8, 9, 10, 11, 12 and 14) were adjusted with dilute acid (hydrochloric acid) or dilute alkaline solution (sodium hydroxide) to get the desired pH solution while other parameters were kept constant.

#### 2.4.6. Effect of extraction time

The extraction of methylphenol was conducted at different times of extraction (5 min, 10 min, 15 min, 20 min, 30 min and 1 h) while other parameters were kept constant.

#### 2.4.7. Effect of interference ions

A series of samples with different types of ions (Li^2+^, Mg^2+^, Br^−^, Cl^−^, $\text{S}{{\text{O}}_{4}}^{2-}$, $\text{N}{{\text{O}}_{2}}^{-}$, $\text{N}{{\text{H}}_{4}}^{+}$, $\text{P}{{\text{O}}_{4}}^{3-}$, F^−^, K^+^, $\text{N}{{\text{O}}_{3}}^{-}$, Na^+^, Ca^2+^) were prepared at 500 ppm concentration in order to investigate the selectivity while other parameters were kept constant.

## 3. Result and discussion

### 3.1. Effect of ionic strength

**Figure 1.**
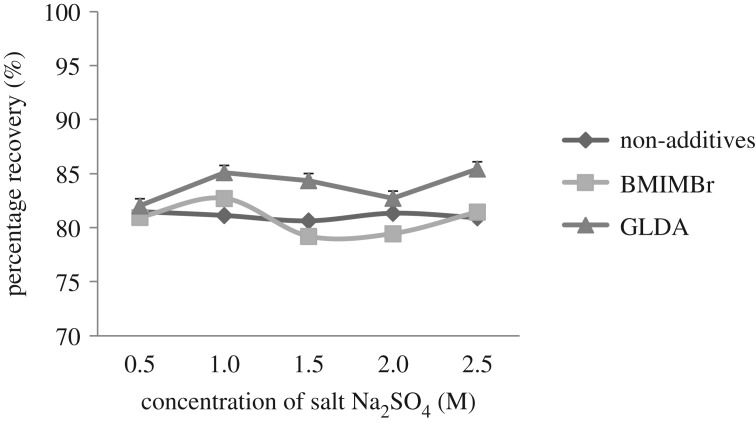
Effect of salt (Na_2_SO_4_) concentration on the percentage recoveries of methylphenol.

Ionic strength is often an important factor for the extraction and enrichment performance of phase separation. The salts increase the density of the aqueous phase [11] and the addition of salts in real water samples may influence the extraction process. Sodium sulphate, Na_2_SO_4_, salt is chosen because it is chemically stable, and does not decompose even when heated [21]. It also does not react with oxidation and reduction agents at normal temperature. Na_2_SO_4_ is a neutral salt with pH 7 when dissolved in water. In this experiment, the concentrations of Na_2_SO_4_ ranging from 0.5 M to 2.5 M were optimized. Figure 1 shows the effects of Na_2_SO_4_ on the CPE method to extract methylphenol from the water samples.

The recovery of methylphenol increased when the salt composition increased and attained optimum value at 1.0 M for the studied additives. The results showed that the electrolyte salt caused an increment in the dehydration of micelles in the surfactant-rich phase. As Na_2_SO_4_ salt acts as a drying agent, it has a very high capacity and efficient capability in controlling the surfactant loss during the CPE method. The salts cause dehydration to occur on both surfactant and methylphenol by breaking the hydrogen bonds with water molecules [21–23]. The recovery decreased at 1.5 M and 2.0 M concentration for all additives. This is because at high concentration salt precipitates appeared at the bottom of the centrifuge tube. However, an increased trend was observed at 2.5 M. Even though the percentage recovery increased, it was not selected as the optimum concentration. This is because at 2.5 M concentration the solution started to precipitate. At high salt concentration, Na_2_SO_4_ molecules were unable to break the hydrogen bonds in the water molecules between the surfactant and methylphenol [22].

As reported by Norseyrihan *et al.* [21], the peak area value of the analytes decreased with increasing concentration of sodium chloride, NaCl [24]. Therefore, 1.0 M salt concentration was adopted as the optimum salt concentration to achieve the best analytical signals and highest extraction recovery for methylphenol extraction.

Previously, Zain *et al*. [15] have investigated the effect of salts in the CPE method including NaCl, NaOH, KCl, KI, Na_2_SO_4_, and K_3_PO_4_ [15]. It was reported that only Na_2_SO_4_ salt could form the two-phase system when the concentration of the salt is in the range of 0.5–1.0 mg l^−1^. However, the other salts are unable to form the two-phase system at a concentration of 2.0 mg l^−1^ or lower. This is because $\text{S}{{\text{O}}_{4}}^{2-}$ ions are cosmotrophic, and exhibit a stronger interaction with water molecules than the water itself [25]. In addition, it is also capable of breaking the water–water hydrogen bonds, which is beneficial to the phase separation [21].

### 3.2. Effect of surfactant concentration

The effects of non-ionic surfactant Sylgard 309 concentration were studied by using a different concentration of surfactant in order to get the maximum enrichment factor that produces a small phase volume ratio [26]. The smaller phase volume ratio of surfactant-rich phase leads to higher concentration of the analyte obtained. The effects of Sylgard 309 surfactant concentration were studied at different concentrations (5%, 10%, 15%, 20%, 30% and 40% (w/v)).

**Figure 2.**
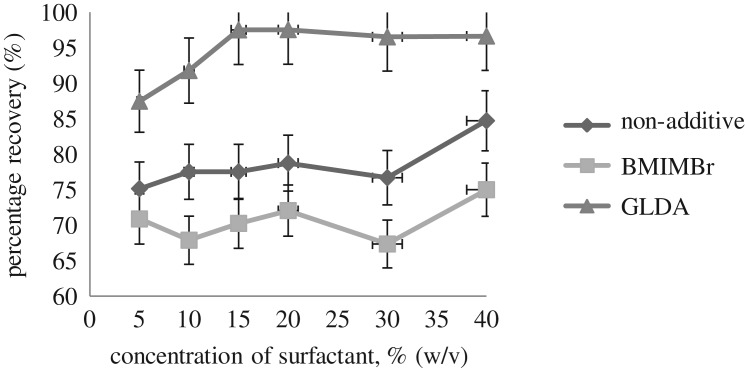
Effect of surfactant concentration on the percentage recoveries of methylphenol extraction.

Figure 2 illustrates the effect of non-ionic surfactant concentration on the extraction recovery of methylphenol. It can be clearly seen that the extraction recovery of methylphenol slightly increased from 10% to 20% (w/v) when the concentration of the surfactant increased for all methods. This was probably due to the increase in the viscosity of the surfactant-rich phase where the viscosity of the surfactant Sylgard 309 would interrupt the CPE phase separation and decrease the volume of the surfactant-rich phase.

At 20–40% (w/v), the recoveries remained practically constant for GLDA but decreased at 20–30% (w/v) for both non-additive and BMIMBr. However, the recoveries for both non-additive and BMIMBr increased dramatically from 30% to 40% (w/v). This is due to the increase of viscosity of the surfactant-rich phase when the concentration of the surfactant increases.

The small volume of the surfactant-rich phase gave a good extraction recovery of methylphenol. The obtained results showed an absolute extraction recovery of methylphenol ranging from 70% to 97% (w/v). This was probably due to the excellent performance of the Sylgard 309 non-ionic surfactant, which was water soluble. This surfactant also has low viscosity for the polyether liquid composition. These properties of the surfactant enhanced the entrapment of the methylphenol in the surfactant-rich phase. Therefore, 15% (w/v) of Sylgard 309 concentration was selected as the optimum surfactant concentration.

### 3.3. Effect of temperature

The optimization of the temperature was studied in order to get the equilibrium temperature in the CPE method. A previous study by Liang & Yang [26] reported that temperature is a significant parameter in the determination of copper in food and water samples using the CPE method [26]. When the CPE method is at equilibrium temperature, the best separation will be achieved [27].

**Figure 3.**
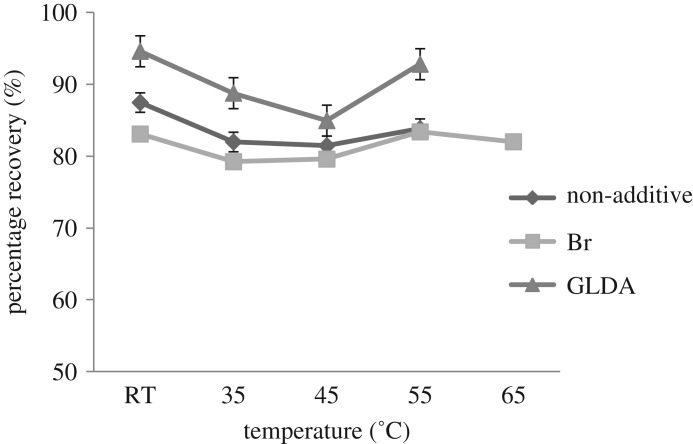
Effect of temperature on methylphenol extraction.

In this study, the desired temperature was maintained between 30°C and 65°C using an ultrasonic water bath with a temperature controller. The results are illustrated in figure 3. The extraction recoveries were in the range of 79–94% and the highest was obtained at 30°C. This result showed that the CPE method is a highly efficient extraction and fast separation method for organic compounds. With no heating process at room temperature, the separation between surfactant and aqueous phase occurred readily. Thus, room temperature was selected as the optimum temperature for methylphenol extraction.

A similar study has been reported by Jalbani & Soylak [6], which concluded that there was also no significant effect of temperature on the extraction recovery of phenol [22]. This is because their results showed a plateau in the extraction recovery of phenol between 20°C and 50°C. From this result, room temperature was taken as the optimum temperature. Thus, the study proved that the temperature of extraction has an insignificant effect on the dehydration of the micelles and the volume of surfactant-rich phase.

### 3.4. Effect of extraction time

The effect of extraction time of methylphenol using the CPE method was studied in the range of 5–60 min. In this study, it was desired to have the shortest extraction time to complete the reaction and an efficient separation of phases. The extraction recovery depends on the time that the analyte has to interact with the micelles and get into their core.

**Figure 4.**
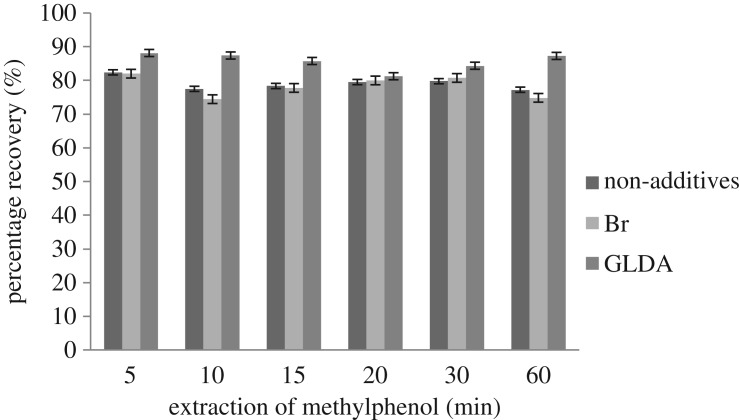
Effect of extraction time on percentage recoveries of methylphenol.

Figure 4 shows the effect of extraction time on the extraction recovery of methylphenol. From the result, it can be clearly seen that the extraction time of 5 min has the highest recovery for all types of additives used. Within 5 min in a thermostatic water bath at 30°C, the salt molecules undergo partial dehydration because the time was sufficient to complete the phase separation between the surfactant-rich phase and the aqueous phase of methylphenol. This occurred by breaking the hydrogen bonds with water molecules of the surfactant and methylphenol, which increased the size of the micelles, thus enhancing the solubility of the analyte [21].

Extraction times of 10 min and longer showed an inconsistent trend of extraction recovery due to the interruption of the salts in the surfactant-rich phase. Hence, 5 min was chosen as the extraction time of methylphenol for the CPE method. Thus, the transfer of analytes from the aqueous phase to surfactant-rich phase was fast and the equilibrium state was quickly achieved [28].

### 3.5. Effect of pH

**Figure 5.**
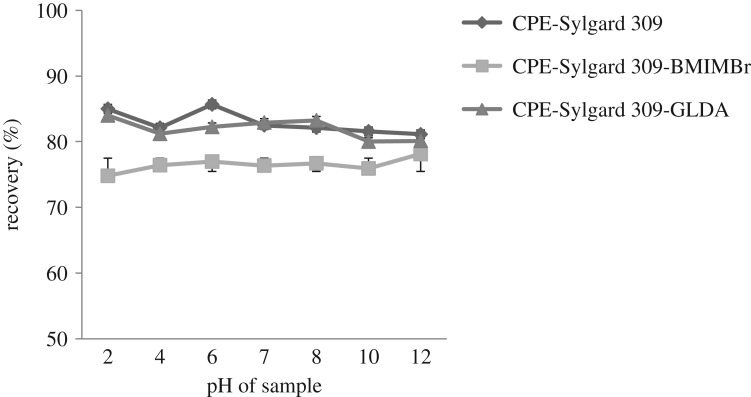
Effect of pH in methylphenol extraction.

The effect of sample pH in the extraction of methylphenol was studied. In the CPE method, the pH is the most important factor regulating the partitioning of the target analytes in the micellar phase for organic molecules. As illustrated in figure 5, the effect of sample pH on the extraction recovery of methylphenol was studied in the range of pH 2 to pH 12.

In figure 5, it is clearly shown that the maximum efficiency for the three additives species was best achieved at a range of pH 6 to pH 12. The extraction efficiencies of the three additives were less in acidic pH and increased as the pH increases.

The extraction recovery of methylphenol was highest at pH 6 for the CPE-Sylgard method because methylphenol exists in neutral form at this condition. This is due to the good interaction between the uncharged methylphenol and Sylgard 309. In highly acidic condition, methylphenol species are protonated and the ionic characteristics of methylphenol in the hydrophobic micelles increase [22], and thus, the effective interaction of methylphenol with Sylgard 309 is restrained in an acidic condition. Xie *et al*. [29] reported that deprotonation of the weak acid or protonation of the weak base results in less strong interaction and binding than for the neutral form with the surfactant aggregate [21,29]. The recovery of methylphenol was at the highest at pH 8 for CPE-Sylgard-GLDA. This may be because of the formation of phenolate ion. Therefore, a range of pH 6 to pH 12 was selected as the optimal pH for the additives in the extraction of methylphenol.

### 3.6. Effect of interference study

The effects of different cations and anions on the extraction of methylphenol in the CPE method were studied. This study was carried out to determine the percentage recovery of methylphenol when some individually spiking ions were added. In this study, an ion was considered to be an interference when it caused an error greater than ±5% [10,30]. To investigate the selectivity of the CPE method, sample solutions containing methylphenol and individually spiking Li^2+^, Mg^2+^, Br^−^, Cl^−^, $\text{S}{{\text{O}}_{4}}^{2-}$, NO^2−^, NH^4+^, $\text{P}{{\text{O}}_{4}}^{3-}$, F^−^, K^+^, NO^3−^, Na^+^ or Ca^2+^ ions were extracted under an optimized CPE with relative standard deviation (RSD) ranging from 0.25% to 1.15%.

**Figure 6.**
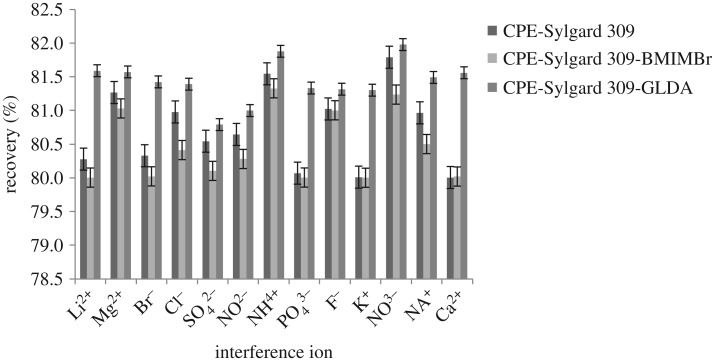
Effect of interference ions in the methylphenol extraction.

Figure 6 shows the effect of selected ions on the extraction of methylphenol. The results revealed that there were no significant changes statistically on the percentage recovery of the methylphenol extraction and none of the ions showed significant interference with the extraction of the methylphenol even when the concentration of each ion achieved 50 ppm. The percentage recovery of the studied methylphenol reached about 80% and above with a 50 ppm concentration of the ions added in the CPE solution. It can be concluded that the method developed is very selective to the parabens compound. Hence, there was no significant interference by the addition of ions present at a moderate concentration.

### 3.7. Water content in the surfactant-rich phase

The water content study was conducted to assess the amount of preconcentration water in the surfactant-rich phase. This study is another factor to be focused on to improve the extraction efficiency. In a study conducted by Zain *et al*. [15], 80 wt% water content was still present in the surfactant-rich phase after the CPE process. According to Bingjia *et al*. [31], the performance of CPE was limited by the water content in the surfactant-rich phase. This is because a lower amount of water in the surfactant-rich phase resulted in a higher concentration of the analyte [31].

**Figure 7.**
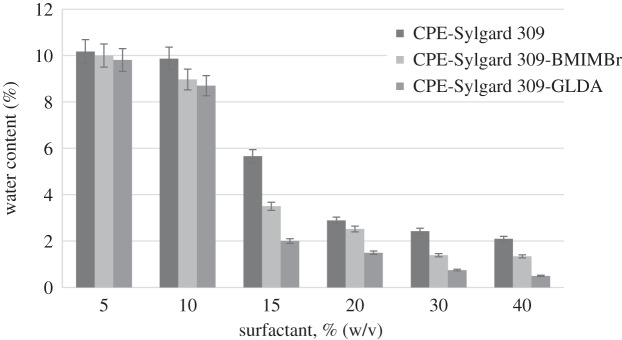
The percentage of surfactant against the percentage of water content.

Figure 7 shows the comparison of the percentage of water content in the surfactant-rich phase between CPE-Sylgard, CPE-Sylgard-BMIMBr and CPE-Sylgard-GLDA. Based on these result, CPE-Sylgard-GLDA obtained the highest percentage of water content in the surfactant-rich phase compared to the other methods.

The CPE-Sylgard-GLDA method obtained the highest amount of water content at the beginning of the experiment with almost 9.81% in the surfactant-rich phase at 5% (w/v) surfactant concentration. This percentage was rapidly reduced to 0.5 at 40% (w/v) surfactant concentration. A reduction of 9.31% was measured for this method. The overall reduction of water content for the other two methods was 8.66% for CPE-Sylgard-BMIMBr and 8.08% for CPE-Sylgard.

**Figure 8.**
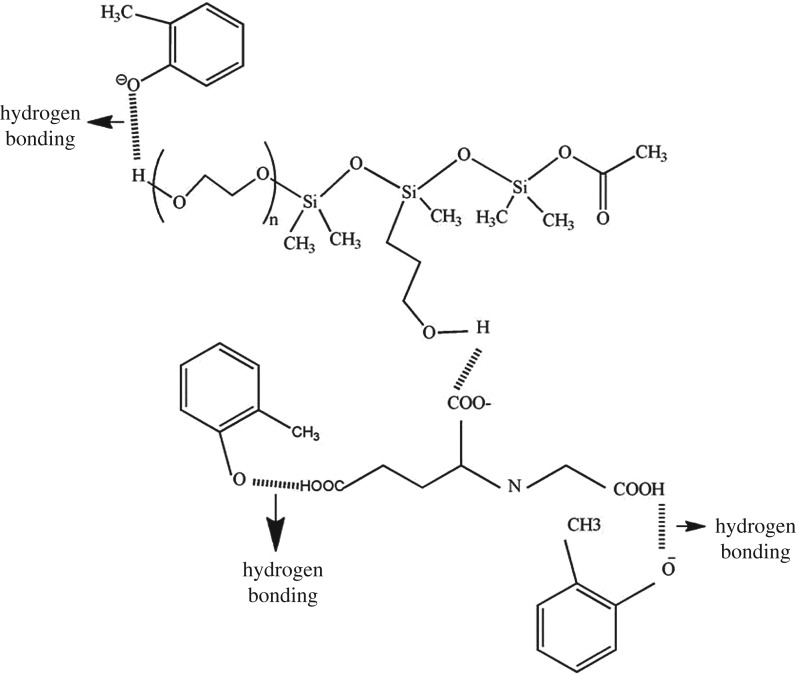
Proposed mechanism of CPE-Sylgard and GLDA interaction.

The CPE-Sylgard-GLDA has the highest water percentage due to the hydrogen bonding in Sylgard-GLDA-methylphenol. From the proposed mechanism based on figure 8, Sylgard and GLDA were combined through hydrogen bonding between the molecules. The methylphenol molecules between both GLDA and Sylgard molecules were also trapped through hydrogen bonding.

In this study, 5% of surfactant concentration used with a range of 0.5% to 2.1% of water content in the surfactant-rich phase was considered low compared to that used in the previous study by Noorashikin *et al.* [21]. The RSD of the water content ranged from 0.02% to 0.06%.

### 3.8. Distribution coefficient

**Figure 9.**
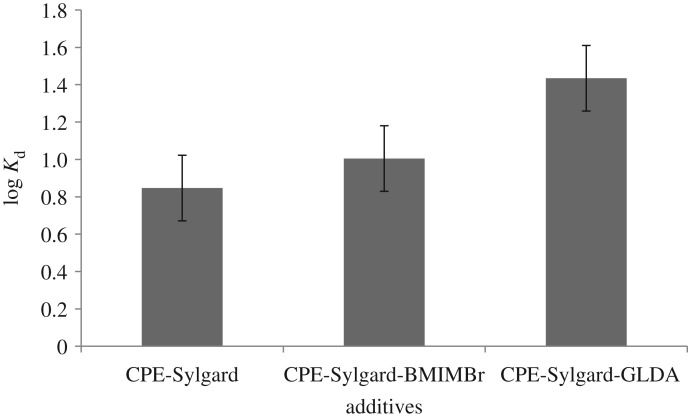
Distribution coefficient of methylphenol.

The distribution coefficient, *K*_d_, is the ratio of methylphenol concentration in the aqueous phase. The distribution coefficient of the three methods was studied and shown in figure 9.

The highest result was obtained from the CPE-Sylgard method. This is because, without an additive such as BMIMBr and GLDA, the methylphenol analyte cannot be absorbed efficiently by the Sylgard 309 into the surfactant-rich phase.

### 3.9. Phase volume ratio on recoveries of methylphenol

**Figure 10.**
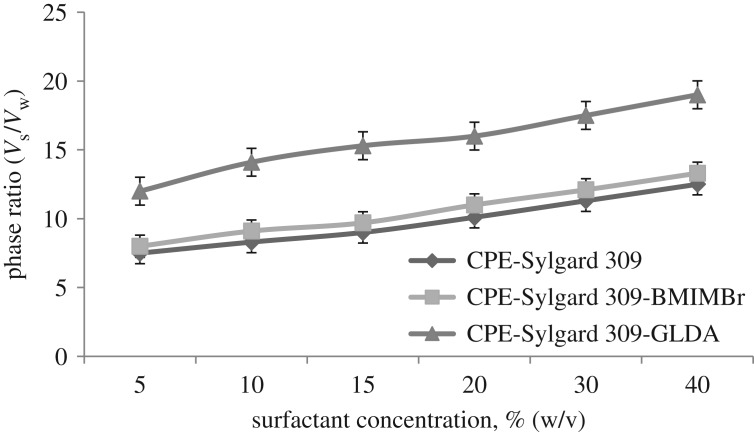
Phase ratio of surfactant concentration for all methods.

The phase volume ratio is the ratio of volume surfactant-rich phase, *V*_s_, to the volume of the aqueous phase, *V*_w_. The plot of *V*_s_/*V*_w_ against the various concentrations of the surfactant is given in figure 10. This plot is important in determining the optimum volume of surfactant-rich phase when CPE is performed. At a lower volume of *V*_s_, the system will produce a higher concentration of methylphenol extracted in the surfactant-rich phase preconcentration factor. However, if the volume of *V*_s_ is too low, it is difficult to extract the methylphenol from the aqueous phase as the amount of surfactant-rich phase is inadequate to work as the efficient extractant.

In figure 10, all methods showed a similar trend whereby when the surfactant concentration increases, the phase volume ratio also increases. This is because when the amount of surfactant increased in the solution, the volume of surfactant-rich phase also increases, resulting in a clear separation between the surfactant-rich phase and aqueous phase.

This is because the addition of additives such as BMIMBr and GLDA used in the system makes the separation easier. The experimental observation showed that when 20% (w/v) surfactant concentration was used for CPE-Sylgard-BMIMBr and 15% (w/v) for CPE-Sylgard and CPE-Sylgard-GLDA, the phase separation between the surfactant-rich phase and the aqueous phase was clearly seen in the centrifuge tube. This surfactant concentration produced low *V*s and was sufficient to extract methylphenol from the aqueous phase. A sufficient and small volume of *V*_s_ was required to produce good extraction recoveries of methylphenol in the real samples.

If a 10% (w/v) surfactant concentration or lower was used in all methods, the volume of *V*_s_ was too small and difficult to separate from the aqueous phase. If a 30% (w/v) or more surfactant concentration was used in those methods, the amount of *V*_s_ was too large resulting in the volume of the surfactant-rich phase being larger and hence less effective in extracting methylphenol from the aqueous phase. It is important to select the optimum phase ratio to ensure that *V*_s_ is sufficient and produces higher percentage recoveries for methylphenol extraction. Therefore, 15% (w/v) surfactant concentration was chosen for the following experiments because in the previous parameter, at 15% (w/v) concentration of surfactant, an optimum recovery was achieved. Figure 8 shows the proposed mechanism of CPE-Sylgard and GLDA interaction.

### 3.10. Method validation

Based on the method described above, the performance of the best method, CPE-Sylgard-GLDA, was tested using real water samples. The relative standard deviations, the coefficient of determination and the percentage recovery are shown in table 1.

**Table 1.** Recovery and concentration of methylphenol in spiked and unspiked water samples.

water samples	recovery (%) and RSD (%) in spiked water samples (*n* = 3)	concentration of methylphenol without spiked water sample, ppm
river water	105.76 (9.85)	0.14
seawater	104.41 (5.54)	0.17
seawater	103.05 (9.51)	not detected
river water	103.59 (5.37)	not detected
river water	107.97 (3.17)	not detected
Mengabang water	108.71 (1.57)	not detected
seawater	99.02 (2.01)	not detected
Mengabang water	107.99 (5.51)	not detected
treated water	98.75 (3.73)	not detected
tap water	99.52 (7.47)	not detected

Water samples were collected from various types of water matrices such as tap water, river water, lake water and seawater in Terengganu and Kelantan, Malaysia. Prior to the analysis, all water samples were successively passed through a 0.45 µm nylon filter to remove any suspended particulate matter and were stored in the dark before extraction. The real water samples of about 0.2 ml were used in the CPE method. The developed CPE method for methylphenol exhibited a better performance with a lower limit of detection (LOD) and limit of quantitation (LOQ) at 0.05 ppm and 0.18 ppm, respectively.

Based on the results obtained in table 1, all real water samples gave excellent percentage recoveries in the range of 97% to 108% with RSD of less than 10%. The results showed a good accuracy of the CPE method and its independence from the matrix effects. The outstanding percentage recovery confirmed the validity of the CPE method on the real water samples.

Mengabang water has the highest percentage recovery because salts in the Mengabang water are entrapped by the CPE method. The addition of salts from the Mengabang water samples enhanced the CPE method. The lowest recovery percentage was obtained for the treated water sample. It was clear that salt in the treated water sample may interrupt the CPE method. These results were similar to a study conducted by Noorashikin and co-workers; the interruption was due to the electrolyte factor of the salt affecting the CPE method [21]. The results indicated an outstanding CPE method, and it was feasible to use this for monitoring methylphenol in environmental water samples [21].

Additionally, higher recoveries were obtained using the developed method of CPE from water samples with unspiked methylphenol. The highest concentration with unspiked methylphenol was extracted from the river water at 0.17 ppm, and 0.14 ppm from the seawater. However, no concentration was detected in the unspiked methylphenol from other samples due to the small concentration of methylphenol. The RSD of unspiked samples was 57.73% for both detected unspiked samples. The result proved that the developed method of CPE is successful for the extraction of methylphenol in the water samples.

Table 2 shows a comparison of the developed method in methylphenol extraction. The current study shows that the developed method is an excellent method to extract parabens compared with the previous method.

**Table 2.** The comparison of the developed method in methylphenol extraction. UV-Vis, ultraviolet visible spectroscopy; HPLC-UV, high performance-liquid chromatography.

	Noorashikin *et al*. [22]	Norseyrihan *et al*. [21]	current study
salt, Na_2_SO_4_ (M)	1.5	2.0	1.0
surfactant concentration	DC193C 30%	Sylgard 309 10%	Sylgard 309 15%
limit of detection	0.076 ppm	0.109 ppm	0.05 ppm ± 0.02 ppm
limit of quantitation	0.46	0.37	0.18
instrument	UV-Vis	HPLC-UV	HPLC-UV

## 4. Conclusion

In this study, the CPE method offers several advantages because it is simple, cost-effective, sensitive, selective, effective and less toxic to the environment. To the best of our knowledge, this is the first report of an optimized method using non-ionic surfactant Sylgard 309 in the CPE method applied for the extraction of methylphenol species in water samples. Experimental results showed that high recoveries can be obtained with the optimized parameters. Furthermore, the non-ionic Sylgard 309 surfactant in the CPE has great potential to be explored for the extraction of organic pollutants in water samples. This is because of the unique structure of the molecules that could entrap hydrophobic as well as hydrophilic substances. In addition, it has a low water content that can enhance the extraction efficiency.

## Supplementary Material

Chromatogram of methylphenol extraction

## Supplementary Material

Chromatogram of methylphenol extraction from spiked real water samples

## Supplementary Material

Chromatogram of methylphenol extraction from unspiked real water samples

## Supplementary Material

Raw data of spiked and unspiked water samples

## Data accessibility

This paper reports primary data. Some data are also presented in the supporting document that has been uploaded as part of the electronic supplementary material.
