# Supplementary figures and images for: Formulation of chelating agent with surfactant in cloud point extraction of methylphenol in water
Source: R Soc Open Sci. 2018 Jul 4;5(7):180070. doi: 10.1098/rsos.180070 (PMC6083667; doi:10.1098/rsos.180070)

Chromatogram of methylphenol extraction


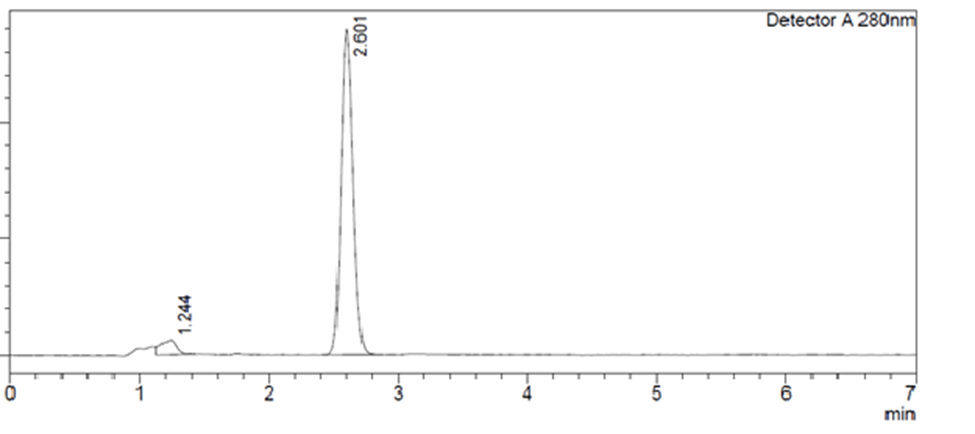


Figure 1 Chromatogram of standard methylphenol

Supplement: Chromatogram of methylphenol extraction [file rsos180070supp1.docx]
