# Supplementary material for: Formulation of chelating agent with surfactant in cloud point extraction of methylphenol in water
Source: R Soc Open Sci. 2018 Jul 4;5(7):180070. doi: 10.1098/rsos.180070 (PMC6083667; doi:10.1098/rsos.180070)
Supplement: Chromatogram of methylphenol extraction from spiked real water samples [file rsos180070supp2.docx]

Chromatogram of methylphenol extraction from spiked real water samples


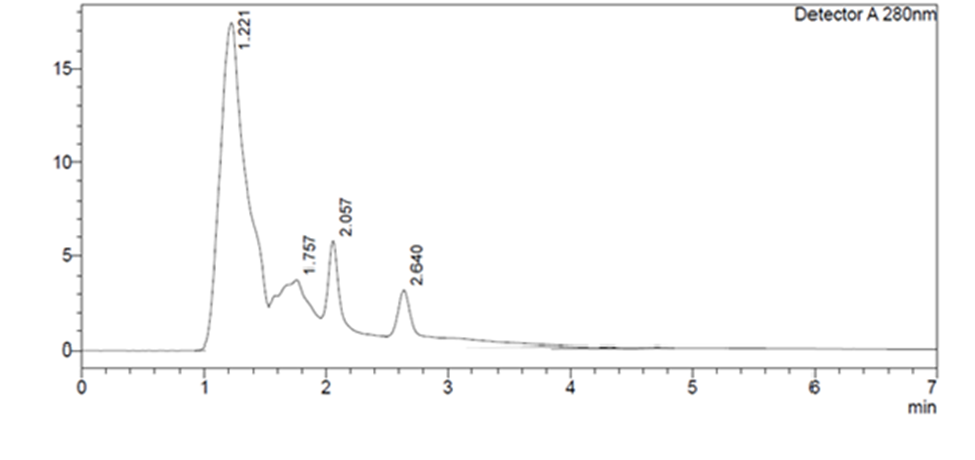


Figure 2 Chromatogram of methylphenol from spiked of real water sample.
