# Supplementary material for: Formulation of chelating agent with surfactant in cloud point extraction of methylphenol in water
Source: R Soc Open Sci. 2018 Jul 4;5(7):180070. doi: 10.1098/rsos.180070 (PMC6083667; doi:10.1098/rsos.180070)
Supplement: Chromatogram of methylphenol extraction from unspiked real water samples [file rsos180070supp3.docx]

Chromatogram of methylphenol extraction from unspiked real water samples

**
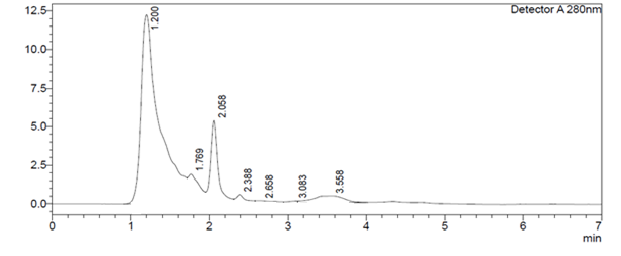
**

Figure 3 Chromatogram of methylphenol from unspiked of real water sample
